# Supplementary material for: HLA alleles measured from COVID-19 patient transcriptomes reveal associations with disease prognosis in a New York cohort
Source: PeerJ. 2021 Oct 15;9:e12368. doi: 10.7717/peerj.12368 (PMC8522641; doi:10.7717/peerj.12368)
Supplement: Supplemental Information 2 [file peerj-09-12368-s002.docx]

**Supplementary data (additionalFile2) for:**

**Warren RL and Birol I. HLA alleles measured from COVID-19 patient transcriptomes reveal associations with disease prognosis in a New York cohort.**

**Supplementary Fig. S1. COVID-19 New-York cohort (Overmyer [27]) transcriptome data characteristics.** Left panel: RNA-Seq read pair count in each patient sample. Right panel: We evaluated base RNA expression across all patient RNA-Seq samples by measuring, using Salmon (Patro et al., 2017), the transcript per million (TPM) expression of house-keeping gene Glyceraldehyde 3-phosphate dehydrogenase (abbreviated GAPDH) (EC 1.2. 1.12) in peripheral blood mononuclear cells (PBMC). This was done solely to evaluate the quality of the RNA-Seq prior to running HLA prediction tools. The average GAPDH TPM is 847.73 +/- 272.32.

Patro R, Duggal G, Love MI, Irizarry RA, Kingsford C. Salmon provides fast and bias-aware quantification of transcript expression. Nat Methods. 2017 Apr;14(4):417-419. doi: 10.1038/nmeth.4197. PMID: 28263959; PMCID: PMC5600148.
